# Frequency of a very brief intervention by physiotherapists to increase physical activity levels in adults: a pilot randomised controlled trial

**DOI:** 10.1186/s13102-019-0118-8

**Published:** 2019-05-22

**Authors:** Nicole Freene, Rachel Davey, Steven M McPhail

**Affiliations:** 1 Physiotherapy, Faculty of Health, University of Canberra, Bruce, 2617 Australia; 2 Centre for Research & Action in Public Health, University of Canberra, Bruce, 2617 Australia; 3 School of Public Health & Social Work and Institute of Health and Biomedical Innovation, Queensland University of Technology, Victoria Park Road, Kelvin Grove, 4059 Australia; 4 Centre for Functioning and Health Research, Metro South Health, Corner of Ipswich Road and Cornwall Street, Buranda, 4103 Australia

**Keywords:** Behaviour change, Fitness, Health status, Accelerometry, Measurement

## Abstract

**Background:**

There is evidence that brief physical activity interventions by health professionals can increase physical activity levels. In addition, there is some evidence that simply measuring physical activity alone can increase physical activity behaviour. However, preliminary work is required to determine the effects of potential measurement frequency. The aim of this pilot study was to examine whether frequency of physical activity measurement, with very brief advice from a physiotherapist, influenced objectively measured physical activity in insufficiently active adults.

**Methods:**

Using concealed allocation and blinded assessments, eligible participants (*n* = 40) were randomised to a lower-measurement-frequency (baseline and 18-weeks) or higher-measurement-frequency group (baseline, 6, 12 and 18-weeks). The primary outcome was daily minutes of moderate-to-vigorous physical activity (accelerometry). Secondary outcomes included functional aerobic capacity (STEP tool), quality-of-life (AQoL-6D), body mass index, waist circumference, waist-to-hip ratio and blood pressure.

**Results:**

Between-group comparisons were not significant in intention-to-treat analyses. However, there was a trend for the higher-measurement-frequency group to complete more daily minutes of moderate-to-vigorous physical activity at 18-weeks (mean difference 19.6 vs − 11.9 mins/week, *p* = 0.084), with a medium effect size (Cohen’s *d* = 0.58). This was significant in per-protocol analysis (*p* = 0.049, Cohen’s *d* = 0.77). Within-group comparisons indicated both groups increased their aerobic fitness (*p* ≤ 0.01), but only the higher-measurement-frequency group decreased their waist circumference (mean decrease 2.3 cm, 95%CI 0.3–4.3, *p* = 0.024), diastolic blood pressure (mean decrease 3.4 mmHg, 95%CI 0.03–6.8, *p* = 0.048) and improved their quality-of-life for independent living (mean increase 3.3, 95%CI 0.2–6.4, *p* = 0.031).

**Conclusion:**

Very brief physical activity interventions by physiotherapists may be an efficient approach to increase physical activity in community-dwelling adults. A larger trial is warranted.

**Trial registration:**

Australian New Zealand Clinical Trials Registry (ANZCTR): ACTRN12616000566437, http://www.ANZCTR.org.au/ACTRN12616000566437.aspx, registered 2 May 2016.

## Background

One quarter of adults worldwide report they are not doing enough physical activity to meet the minimum recommended guidelines for health benefits, and in Australia, the country of this study, the situation is less favourable, with approximately half of adults reporting insufficient levels of physical activity [1, 2]. Internationally there is a focus on the development and refinement of physical activity interventions as insufficient physical activity is a major risk factor for chronic disease and death [3–6]. Physical activity promotion by health professionals is one type of physical activity intervention that is viewed as a key strategy to improve the populations’ physical activity levels. This strategy has been outlined in action plans around the world, and there is some evidence that brief physical activity interventions by health professionals are just as effective as more intensive interventions [7–10].

Health professionals, such as physiotherapists, are well placed to promote and assess physical activity. Physiotherapists are effective communicators, establishing rapport, gaining trust, supporting and empowering an individual [11]. Physiotherapists perceive the provision of physical activity advice as part of their role, and there is evidence that physiotherapists can counsel effectively for physical activity behaviour change and also treat any underlying conditions that may impair someone’s physical capacity to be physically active [12–14]. Kunstler et al. (2017) found in a systematic review that adults receiving a physiotherapist-led physical activity intervention in private practice, primary care or outpatient settings doubled their odds of increasing their physical activity levels up to 1 year after the intervention. Yet, internationally it appears that physical activity levels are not routinely assessed by physiotherapists and brief physical activity interventions are not routinely delivered in clinical practice, with lack of time reported as the most commonly perceived barrier [13, 15, 16].

Interestingly, prior research has indicated simply measuring physical activity levels in control groups, as part of randomised controlled studies investigating physical activity interventions, has been found to increase physical activity behaviour [17]. Waters et al. (2012) found that approximately one third of physical activity intervention studies in primary care have reported improvements in physical activity among participants who were in the control group. In fact, some control groups have been found to increase their physical activity levels to a similar level as the intervention group, particularly over the longer term [18, 19]. Measurement reactivity, the Hawthorne effect, higher intensity of contact compared with a clinical setting and motivated volunteers are possible explanations for these findings [17, 20, 21]. This may indicate with minimal contact and resources, physical activity behaviour change may be achievable in a proportion of the population. Although, of the 29 studies included in the Waters et al. (2012) systematic review, only four measured physical activity objectively (accelerometry), and this was not included as an outcome in this review. Similarly, Lamming et al. (2017) in their systematic review of reviews found that brief interventions (less than 30 min) can increase self-reported physical activity in the short-term (4–12-weeks), and they recommended that future research should focus on very brief interventions (less than 5 min) [22]. Thus, frequent measurement of physical activity, with very brief advice by health professionals, in routine clinical care may be a very brief intervention that can result in favourable increases in physical activity levels.

With the ever increasing access to objective activity monitoring devices (accelerometers in smartphones, wrist, shoe or arm worn devices), combined with the broad reach of health professionals, even a modest effect of measurement and very brief advice, with accountability to their health professional, may prove to be an efficient and effective approach to increase the populations’ physical activity levels in a meaningful way. However, preliminary work is required to determine the effects of potential measurement and very brief advice frequency. The aim of this study was to examine whether frequency of physical activity measurement and very brief advice provided by physiotherapists increased physical activity levels in a sample of insufficiently active Australian adults.

## Methods

A pilot (two parallel arm, 1:1 allocation ratio) randomized control study was conducted between July and November 2016. Participants were recruited to two, or four, health and fitness assessments at an outpatient clinic using magazine, electronic media and poster advertising. All individuals that responded to the advertisements were contacted by email or telephone by the principal researcher to determine their eligibility. Eligible participants were between 18 and 64 years old, insufficiently active (less than 150 min of self-reported moderate-to-vigorous physical activity (MVPA) per week), had no serious medical conditions that could limit participation in moderate physical activity, no severe functional impairments due to medical and psychiatric conditions, and adequate English and cognitive skills to participate in the study. Only one person per household was eligible and participants were not planning to move from the city within the 18-week study period. Medical clearance screening was undertaken using the Sports Medicine Australia Pre-Exercise Screening System [23]. All participants provided written consent prior to completion of their baseline assessment.

An investigator who was at a remote location and not involved in recruitment or assessments used a computer to generate a random number sequence and concealed group allocation using sealed, consecutively numbered opaque envelopes. Following each participant’s baseline assessment the next envelope in the sequence was opened to reveal random allocation to one of two groups: lower-measurement-frequency (LMF; 2 physiotherapist-led physical activity measurements at baseline and week 18) or higher-measurement-frequency (HMF; 4 physiotherapist-led physical activity measurements at baseline, week 6, 12 and 18).

### Very brief physical activity intervention

At all assessments participants were given an indication of their fitness, that is, predicted maximum oxygen consumption (VO_2_max) based on the Step Test and Exercise Prescription (STEP) tool [24]. At baseline, participants received two brochures ‘Make your Move – Sit less – Be active for life!’ [25] and ‘Healthy Eating for Adults’ [26]. The physical activity guidelines were briefly discussed (2 min) at each assessment but participants did not receive any other advice or support for increasing exercise or physical activity levels. Examples of the brief physical activity advice provided are ‘moderate intensity physical activity means you should be able to talk in full sentences but not sing’, and ‘30 min of moderate physical activity can be accumulated in 10 min bouts’. All participants were encouraged to increase their physical activity levels safely during the 18-week period aiming to progress towards the public health physical activity guidelines, that is, accumulate 150 to 300 min of moderate intensity physical activity or 75 to 150 min of vigorous intensity physical activity or a combination of both each week [27]. The total time required for the STEP tool (mean test length = 70 ± 15 s) and provision of brief advice was less than 5 min, classifying it as a very brief intervention [28].

### Outcome measures

The assessor at baseline and 18 weeks for all participants was blinded to group allocation. To maintain blinding, a different assessor was used for the week 6 and week 12 assessments for the higher-measurement-frequency group (which were not analysed). The higher-measurement-frequency assessor was trained in all measurement procedures, risk communication and brief physical activity advice to ensure standardisation, although one assessor may have been more supportive than the other.

A triaxial commercial accelerometer (ActiGraph GT3X or ActiSleep, Fort Walton Beach, FL) was used to objectively assess physical activity. Participants were given the accelerometer during each assessment and asked to wear the monitor on their right hip, while awake, for 7-consecutive days, retuning the accelerometer via mail in the reply-paid post pak provided. All data was downloaded and screened, excluding data if: < 10 h per day wear time (non-wear defined as > 60 consecutive minutes where there is zero activity, allowing for 2 min of counts between 0 and 100) and less than 4 days of valid data. The raw data collected by the accelerometer, counts, was then used to obtain the time spent in different physical activity intensities. The Freedson Combination energy expenditure algorithm was used to determine the physical activity intensity cut-points [29]. MVPA bout data used a minimum bout length of 10 min, allowing for 2 min of counts less than the MVPA threshold within this time. Estimating time spent in physical activity was calculated by dividing the total time spent (minutes) in each threshold by the number of valid days. Adherence to the physical activity recommendations (sufficient time) was calculated for 1 s epoch and 10 min bout data [27]. Calculations were based on estimates of daily minutes in MVPA, weighting vigorous physical activity by 2 for 1 s epoch data, and multiplying by 7 to estimate a week.

The Active Australia Survey (AAS) has been designed to measure participation in leisure time physical activity and to assess the participant’s knowledge of current public health messages about the health benefits of physical activity [30]. It offers a short and reliable set of questions and applies to 1 week preceding the interview, including walking for transport. The AAS has evidence to support its reliability and validity [31, 32].

The STEP tool was used to assess functional aerobic capacity. The STEP tool is an indirect measure of aerobic capacity, requiring a set of 2 × 20 cm steps and is valid for use with adults aged 18–85 years [24, 33]. Participants step up and down a standardized set of 2 steps, 20 cm each, 20 times at a self-selected normal pace following a demonstration of the test. Time to complete the test (secs) and post-test heart rate (bpm) are recorded, calculating predicted VO_2_max (ml/kg/min) using these measures and age (yrs), sex, and body weight (kg); VO_2_max = 3.9 + (1511/time)*((weight/HR)*0.124)-(age*0.032)-(sex*0.633) [24]. Normative values for classifying VO_2_max (poor-superior) were used to give the participant an indication of their aerobic fitness [24].

The Assessment of Quality of Life (AQoL)-6D is a health-related quality of life questionnaire that was self-administered. The AQoL-6D has 20 items with 4–6 levels, takes 2–3 min for participants to complete, and has six dimensions - Independent Living, Mental Health, Coping, Relationships, Pain, Senses, as well as an overall multi-attribute utility score that can be derived from AQoL responses. The AQoL-6D was scored using the recommended Algorithm for Adults, where a score of 100 reflects best health [34]. The AQoL has evidence supporting its reliability and validity in community settings [35, 36].

Height (m), weight (kg) and body mass index (BMI, kg/m^2^) were recorded using a calibrated set of scales and a stadiometer. Waist circumference and hip circumference were measured in centimetres using a tape measure, and waist-to-hip ratio was calculated. Resting blood pressure was measured using a mercury sphygmomanometer on the right arm of seated subjects. Sociodemographic information was also collected with questions regarding participant’s education level, relationship status, current employment status and the presence of any chronic diseases.

### Sample size

An important purpose of this study was to provide an estimate of the potential effect size of measuring physical activity at two different frequencies over an 18-week period, and there was no prior appropriate data assessing the change in physical activity levels using accelerometey on which to base a sample size estimate for between group comparisons [37]. Therefore, the study sought to recruit 40 participants for this purpose. Data from this study will be used to inform planning for future studies.

### Data analysis

Analyses were carried out following intention-to-treat principles. As a conservative approach for missing data at 18-weeks, no change from baseline was assumed for the intention-to-treat analyses. In addition, a less conservative approach (per-protocol analysis) was completed using only participants who had completed scheduled assessments. Descriptive analyses were also completed. Normality of the data was assessed using the Kolmogorov-Smirnov test. For between group analyses, unpaired t-tests were used for data that was normally distributed, the Mann-Whitney U test was used for data that was not normally distributed, and Chi-squared analysis was used for categorical data. For within group analyses, changes in variables with normal distributions were analysed with paired t-tests, if variables were not normally distributed the Wilcoxon Signed Ranks tests were used, and the McNemar test was used for changes in proportions. All analyses were conducted using SPSS version 23. Significance level was set at *p* < 0.05.

## Results

**Fig. 1:**
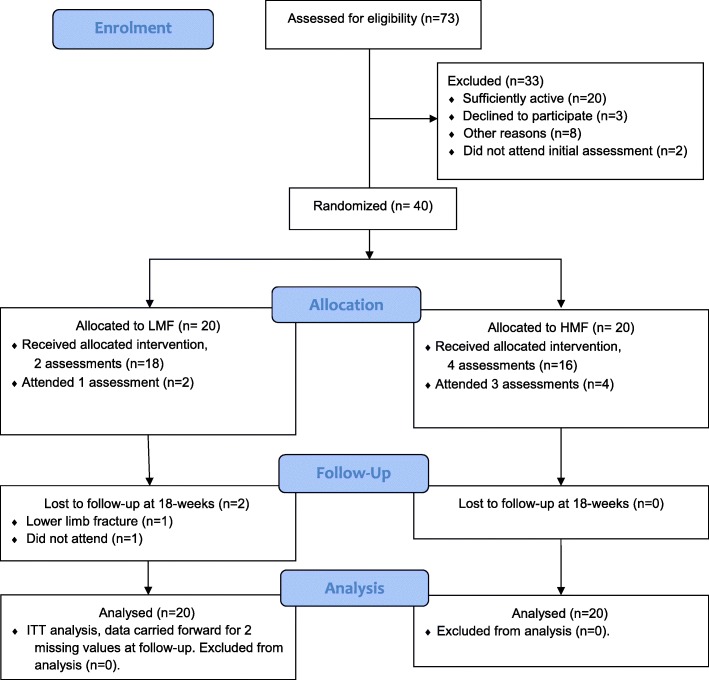
Flow of participants. HMF, higher-measurement-frequency, 4 physical activity measurements and very brief advice (intervention); LMF, lower-measurement-frequency, 2 physical activity measurements and very brief advice (control); ITT, intention-to-treat

Forty participants were recruited (Fig. 1), with a mean age of 44 years, and age range from 20 to 63 years old. Participant characteristics are reported in Table 1. At baseline, there were no differences between groups in objectively measured physical activity. Physical activity measurements are reported in Table 2. Measures of disease risk, fitness and health-related quality of life are reported in Table 3.

**Table 1:** Participant characteristics at baseline

Characteristic	Baseline	
	HMF	LMF
Age in years, mean (SD)	46 (14)	41.5 (14.8)
Gender, n females (%)	17 (85)	16 (80)
Country born, n Australia (%)	16 (89)	16 (80)
Employment, n paid work (%)	12 (71)	12 (63)
Education level, n tertiary (%)	15 (83)	16 (80)
Relationship status, n partner (%)	12 (67)	16 (80)
Chronic disease, n no (%)	15 (83)	20 (100)
Blood pressure medication, n no (%)	16 (89)	19 (95)

HMF, higher-measurement-frequency, 4 physical activity measurements and very brief advice (intervention); LMF, lower-measurement-frequency, 2 physical activity measurements and very brief advice (control)

**Table 2:** Physical activity characteristics at baseline and 18-weeks

Characteristic	Baseline		18-weeks	
	HMF	LMF	HMF	LMF
MVPA minutes/day, mean (SD)				
Actigraph 1 s	64.7 (31)	53.7 (20.2)	68.4 (33.7)	52.6 (18.7)
Actigraph 10 min bout	15.4 (19.1)	13 (10.3)	19.5 (31.2)	10.1 (10.8)
Self-report (AAS)	49 (37.2)	44.5 (38.8)	51.4 (42.5)	40.1 (27.3)
Sufficient PA time, n (%)				
Actigraph 1 s^#^	18 (100)	18 (100)	19 (100)	19 (100)
Actigraph 10 min bout	3 (16.7)	3 (16.7)	4 (21.1)	3 (16.7)
Self-report (AAS)^#^	15 (83.3)^A^	15 (75)	13 (68.4)	14 (73.7)
VM counts/day, mean (SD)	587,087 (223086)	511,455 (131679)	615,223 (278145)	504,438 (138266)

^A^unpaired comparison between groups, *p* ≤ 0.01; ^#^vigorous physical activity multiplied by 2. *HMF* higher-measurement- frequency, 4 physical activity measurements and very brief advice (intervention), *LMF* lower-measurement-frequency, 2 physical activity measurements and very brief advice (control), *MVPA* moderate-to-vigorous physical activity, *AAS* Active Australia Survey, *PA* physical activity, *VM* accelerometer vector magnitude

**Table 3:** Measures of disease risk and fitness at baseline and 18-weeks

Characteristic	Baseline mean (SD)		18-weeks mean (SD)	
	HMF	LMF	HMF	LMF
Waist circumference (cm)	84.7 (12.6)	85.2 (15.1)	82.3 (11.5)^A^	84 (13)
Waist-to-hip ratio	0.79 (0.08)	0.81 (0.09)	0.79 (0.08)	0.80 (0.8)
Body mass index (kg/m^2^)	28.1 (5.4)	26.4 (4.1)	28 (5.2)	26.5 (4.2)
SBP (mmHg)	126 (10)^B^	119 (9)	123 (9)	119 (10)
DBP (mmHg)	82 (10)	77 (9)	78 (7)^A^	76 (7)
AQoL-6D utility score	82.9 (7.9)	84.8 (7.9)	84.4 (6.7)	85.9 (5.8)
AQoL-6D Independent Living	95 (9.2)	94.8 (6.4)	98.3 (4.5)^A^	99.3 (2.5)
AQoL-6D Relationships	93 (8.7)	90.5 (13.2)	94.5 (6.9)	92.9 (7.1)
AQoL-6D Mental Health	74 (14)	71.9 (13.7)	74.4 (11.8)	73.2 (11.9)
AQoL-6D Coping	72 (13)	75 (12.7)	72.5 (10.9)	76.6 (8)
AQoL-6D Pain	79.6 (21.4)	85.5 (19.6)	79.5 (26.5)	89.7 (18)
AQoL-6D Senses	81.9 (10.4)	86.5 (10.8)	84.2 (9.2)	83.3 (8.7)
STEP predicted VO_2_max (ml/kg/min)	40.2 (11.9)	45.1 (8.9)	43.6 (13.3)^C^	47.5 (10.8)^C^

^A^paired comparison within group, *p* < 0.05; ^B^unpaired comparison between groups, *p* < 0.05; ^C^paired comparison within group, *p* ≤ 0.01. *HMF* higher-measurement- frequency, 4 physical activity measurements and very brief advice (intervention), *LMF* lower-measurement-frequency, 2 physical activity measurements and very brief advice (control), *SBP* systolic blood pressure, *DBP* diastolic blood pressure, *AQoL-6D* Assessment of Quality of Life 6 Dimension questionnaire, *STEP* Step Test and Exercise Prescription tool, *VO*_*2*_*max* maximum oxygen consumption

At 18-weeks 95% (38/40) of participants completed assessments (Fig. 1). Thirty-six participants provided valid accelerometer results at baseline, and 30 participants had valid accelerometer results at 18-weeks. There was no significant difference between groups for accelerometer wear time, with participants wearing the accelerometers for approximately 14.5 h per day, for a mean of 6.9 days.

The intention-to-treat analysis indicated there was no significant difference between or within groups in physical activity levels, both self-reported and measured with the accelerometer, at 18-weeks (Table 2). However, there was a trend for participants in the higher-measurement-frequency group to complete more accelerometer daily minutes of MVPA than the lower-measurement-frequency group at 18-weeks, with a medium effect size (Table 2, *p* = 0.084, Cohen’s *d* = 0.58). This corresponded to a mean increase of 19.6 MVPA minutes/week in the higher-measurement-frequency group versus an 11.9 MVPA minutes/week decrease in the lower-measurement-frequency group. This finding was significant in the per-protocol analysis comparison between groups at 18-weeks, with the higher-measurement-frequency completing significantly more accelerometer daily minutes of MVPA (1 s) than the lower-measurement-frequency group (*p* = 0.049, Cohen’s d = 0.77).

Both groups significantly increased their aerobic fitness (*p* ≤ 0.01). Although, only participants who were in the higher-measurement-frequency group significantly decreased their waist circumference (mean decrease 2.3 cm, 95%CI 0.3–4.3, *p* = 0.024), diastolic blood pressure (mean decrease 3.4 mmHg, 95%CI 0.03–6.8, *p* = 0.048) and improved their quality of life independent living domain (mean improvement 3.3, 95%CI 0.2–6.4, *p* = 0.031). There were no adverse events recorded.

## Discussion

This is the first randomised trial to report the effect of frequency of physical activity measurement and very brief advice by physiotherapists on objective physical activity levels. These preliminary findings suggest that frequent physical activity measurements with very brief advice by physiotherapists may result in increased objectively measured physical activity. This may also be associated with improvements in other health outcomes such as decreasing waist circumference and diastolic blood pressure, and improving quality-of-life. Importantly, regardless of physical activity measurement frequency, participants significantly increased their aerobic fitness. Further research is indicated to confirm these findings.

van Sluijs et al. (2006) compared different frequencies of physical activity measurement in a large randomised controlled study in Dutch general practice (*n* = 635). Participants were randomised to 3 or 1 physical activity measurements over 6 months. More participants in the 3 physical activity measurement group met the physical activity guidelines at the end of the intervention period as compared to the 1 physical activity measurement group when considering self-reported physical activity but there was no difference found between groups for a sub-sample of participants using accelerometry. The authors concluded that the increased frequency of physical activity measurements affected participants’ physical activity behaviour. However, these findings ought to be interpreted cautiously as there was limited self-reported physical activity data collected at baseline for comparison and no baseline objective measurement of physical activity. The present study has extended the field by reporting for the first time a trend for an increase in both subjective and objective measurement of physical activity in the higher-measurement-frequency group (Table 2).

Consideration must be given to the length of the physical activity intervention if it is to be feasible in a health care setting. Lack of time is consistently reported as a barrier to physical activity promotion by physiotherapists [38]. For physical activity promoting interventions to be translated into clinical practice, they must be brief, whether or not they include measurement [22]. This study has reported on a very brief intervention including measurement of functional aerobic capacity with feedback and physical activity advice, recognising that physical fitness and physical activity are closely linked and are predictors of all-cause mortality and cardiovascular events [39, 40].

There are important caveats that need to be considered when interpreting findings from the present study. Very brief physical activity interventions, or physical activity measurement alone, may require a longer intervention period to result in changes in physical activity behaviour. According to social cognitive theory, for an increase in physical activity to be adopted and maintained it must be sustained for at least 6-months [41]. Thus, the 18-week time period used in this study may not have been of sufficient length to allow for sustained changes in physical activity behaviour. In addition, Waters et al. (2012) and Opendeacker et al. (2011) found that control group changes in physical activity were more likely when follow-up assessments were carried out over a longer period of time. Waters et al. (2012) found in their systematic review that follow-up assessments completed at 9 months as compared to 7 months were more likely to result in a clinically meaningful improvement in physical activity in control groups. Opendecker et al. (2011) found at 2-years, with a 12-month no intervention follow-up, there was no difference in aerobic fitness between 2 intervention groups (structured vs lifestyle) and a control group, concluding this was consistent with the control group improvement in physical activity and this was possibly due to a measurement effect. Therefore, future physical activity measurement studies should consider measuring physical activity over a 6-month or greater period according to the social cognitive theory and improvements of physical activity in control groups.

Uncertainty remains about the most beneficial physical activity intervention components in adults, in terms of intervention length, intensity and mode, for example, individual versus group-based, face-to-face versus remotely delivered [42, 43]. What is known is that most physical activity interventions work while they are being conducted, with positive results unlikely to be sustained over the longer term [44]. Professional background of those who deliver the physical activity intervention does not seem to influence the outcome but frequency of contacts might [42]. Considering the reach of health professionals around the world, further research is indicated to determine if an increased frequency of very brief physical activity interventions can increase adults’ physical activity levels. If very brief physical activity interventions by physiotherapists are found to be successful, it may be a low cost efficient and effective method to increase a proportion of the populations’ physical activity levels, leading to improved health throughout adulthood and into older age.

### Limitations and strengths

There are many strengths of this pilot study such as the use of randomisation, concealed allocation, assessor blinding, objective measurement of physical activity, all participants being assessed over the same time period to eliminate a seasonal effect and use of intention-to-treat (primary) analyses, as well as a (secondary) per-protocol analysis for objectively measured physical activity. However, findings from this study should be interpreted with caution as the sample size was small, valid final accelerometer results were only provided by 75% (*n* = 30/40) of the sample and the participants were predominantly educated women in a relationship, and dissimilar samples may not have responded in the same way. A medium effect size was found for the higher-measurement-frequency group which is useful for future sample size calculations. Although, it is unclear whether it was the additional physical activity measurements or the additional contacts with the physiotherapist that may have made a difference to physical activity levels. Furthermore, the intervention appeared to be feasible, requiring little time to implement (approximately 5 min for the aerobic fitness measure and physical activity advice), and limited space and equipment.

## Conclusion

Frequency of physical activity measurement with very brief advice by physiotherapists may be enough to improve health outcomes in insufficiently active community-dwelling adults. Further research is indicated with larger sample sizes and longer follow-up to determine whether increased frequency of very brief physical activity interventions by physiotherapists results in increased physical activity levels in community-dwelling adults.

## Abbreviations

AAS: Active Australia Survey
AQoL-6D: Assessment of Quality of Life 6 Dimension questionnaire
BMI: Body mass index
HMF: Higher-measurement-frequency
LMF: Lower-measurement-frequency
MVPA: Moderate-to-vigorous physical activity
STEP: Step Test and Exercise Prescription
VO_2_max: Maximum oxygen consumption
